# Arthroscopic Optical Coherence Tomography in Diagnosis of Early Arthritis

**DOI:** 10.1155/2011/671308

**Published:** 2011-04-03

**Authors:** Michael J. O'Malley, Constance R. Chu

**Affiliations:** Department of Orthopaedic Surgery, Cartilage Restoration Center, University of Pittsburgh Medical Center, BST 1640 200 Lothrop Street, Pittsburgh, PA 15213, USA

## Abstract

Osteoarthritis (OA) is a progressive, debilitating disease that is increasing in prevalence. The pathogenesis of OA is likely multifactorial but ultimately leads to progressive breakdown of collagen matrix and loss of chondrocytes. Current clinical modalities employed to evaluate cartilage health and diagnose osteoarthritis in orthopaedic surgery include, radiography, MRI, and arthroscopy. While these assessment methods can show cartilage fissuring and loss, they are limited in ability to diagnose cartilage injury and degeneration prior breakdown of the articular surface. An improved clinical ability to detect subsurface cartilage pathology is important for development and testing of chondroprotective and chondrorestorative treatments because the pathological changes following surface breakdown are generally considered to be irreversible. Optical Coherence Tomography (OCT), is a novel, non-destructive imaging technology capable of near-real time cross-sectional images of articular cartilage at high resolutions comparable to low power histology. This review discusses a series of bench to bedside studies supporting the potential use of OCT for enhanced clinical diagnosis and staging of early cartilage injury and degeneration. OCT was also found to be useful as a translations research tool to assist in clinical evaluation of novel quantitative MRI technologies for non-invasive evaluation of articular cartilage.

## 1. Introduction

Osteoarthritis (OA) affects over 27 million adults in the United States today, and the prevalence is expected to increase to 67 million by 2030 [1]. The pathogenesis of osteoarthritis is likely multifactorial involving mechanical, biological, biochemical, and genetic factors [1–4]. These factors can all contribute to progressive degeneration and loss of articular cartilage. In the earliest stages of cartilage injury and degeneration, proteolytic breakdown of the extracellular matrix, which is comprised primarily of collagen type-II and glycosaminoglycans, occurs [2–4]. In addition, there may also be actual or functional loss of articular chondrocytes. The remaining healthy chondrocytes attempt to balance the formation and breakdown of matrix molecules. However, the balance between anabolic and catabolic processes ultimately exceeds the repair capabilities of the chondrocytes resulting in matrix destruction, cartilage loss, and eventually, osteoarthritis [3, 4].

Current clinical modalities employed to evaluate cartilage health in orthopaedic surgery include radiography, MRI, and arthroscopy. Unfortunately, these assessment methods cannot reliably diagnose early cartilage injury and degeneration prior to loss of articular cartilage surface integrity, following which the pathological changes may be irreversible [4]. Experimental evidence shows that chondrocyte metabolic deficits occurring prior to breakdown of the articular surface may be reversible [5]. As such, clinically useful methods to detect subsurface cartilage injury and degeneration are important for development and testing of chondroprotective and chondrorestorative therapies. Optical Coherence Tomography (OCT) is a novel, nondestructive imaging technology capable of near-real-time cross-sectional imaging of articular cartilage at high resolutions comparable to low power histology [6–11]. The following describes the advent of OCT for arthroscopic imaging of articular cartilage and the potential use of OCT as a new clinical tool for enhanced clinical diagnosis and staging of early cartilage injury and degeneration.

## 2. Current Clinical Imaging Modalities

Minimally invasive arthroscopic imaging of the articular cartilage is considered the clinical standard for detection of early cartilage injury and degeneration. During arthroscopy, the cartilage is graded from 0 to 4 using the Outerbridge scoring system (0 = firm cartilage, 1 = softening, 2 = fissuring of <50% of cartilage thickness, 3 = fissuring >50% of cartilage thickness, and 4 = exposed bone) [12]. Arthroscopy is primarily a surface imaging technology combined with subjective tactile probing. As such, arthroscopy falls short of the laboratory assessment standards of histopathology, metabolic study and biomechanical testing. Experimentally, biopsy and histology can detect matrix degradation and structural breakdown in cartilage that exhibits no gross surface abnormalities when observed by arthroscopy [7]. However, this is not a practical means for routine clinical detection of early arthritis since histology requires removal and destruction of the tissue being examined. Historically, radiographs were used to diagnose osteoarthritis. However only end-stage bone-related changes are reliably detectable by radiographic exam which does not adequately show soft tissues or directly image articular cartilage. MRI, while being a noninvasive cross-sectional imaging technology, suffers from low resolution and the inability of standard MRI to discern matrix changes leading to cartilage “softening” [13]. As such, arthroscopy remains the current clinical standard for diagnosis and staging of early articular cartilage injury and degeneration.

## 3. Optical Coherence Tomography

**Figure 1:**
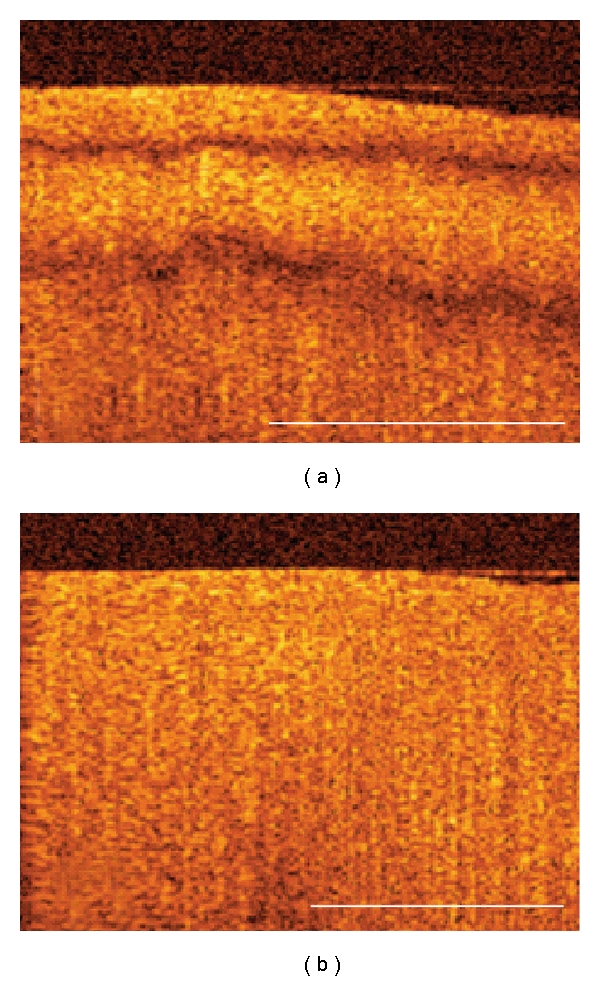
Cartilage OCT form birefringence. (a) OCT image of cartilage with OCT form birefringence where distinct dark bands create a multilayered appearance. (b) OCT image of cartilage without OCT birefringence. In cartilage graded to be without OCT form birefringence, there were no recognizable banding patterns in any of the four scan orientations. Scale bar = 1 mm.

Optical Coherence Tomography (OCT) is a novel imaging modality that allows for a nondestructive, cross-sectional “optical biopsy” of tissue [10, 14]. The technology has been well described and is currently used clinically by ophthalmology to image the cornea and retina and is used experimentally to image coronary arteries and malignancies [6, 15–17]. At a very basic level, Optical Coherence Tomography can be considered similar to ultrasound except that OCT uses infrared light instead of sound waves [6, 10]. The backscatter of light reflected by the tissue is detected and filtered such that only coherent waves are processed by the OCT system producing an ultra high-resolution (4–20 *μ*m) digital image on a computer screen (Figure 1) [5, 6, 10, 18–20]. In early OCT systems, the measurement of time-of-flight by the optical signal allows for production of a two-dimensional image and detection of spatial relationships between adjacent structures [10]. This OCT technology is referred to as time-domain OCT because the image production and resolution is based on a function of distance traveled over time by the infrared light signal. In contrast, spectral-domain OCT detect differences in tissue composition based on changes in the frequency of backscattered light allowing for more efficient data acquisition and faster scan speeds producing near-real-time images [16, 21, 22]. In addition, polarization-sensitive OCT (PS-OCT) is another OCT technology that differs again by containing the ability to detect changes in the polarization state of the backscattered light permitting quantification of tissue birefringence. 

## 4. Optical Coherence Tomography of Articular Cartilage

Articular cartilage exhibits natural birefringence that is detectable by light microscopy due to the organization of its collagen fibrils [21]. Herrmann et al. showed that normal cartilage is sensitive to the polarization state of the incident light of OCT and that the cartilage birefringence could be evaluated using polarization sensitive OCT [9]. Early in the progression of osteoarthritis, collagen fibrils of articular cartilage become disorganized. Drexler et al. examined the relationship between the polarization sensitivity of cartilage as detected by OCT and the changes seen in collagen organization as determined by polarized microscopy of human osteochondral explants [23]. They determined that collagen disorganization found in arthritic articular cartilage as detected by polarized microscopy is detectable by PS-OCT as a loss of normal form birefringence [23].

Using a polarized fiberoptic OCT system, Chu et al. evaluated healthy and degenerating cartilage in grossly normal appearing human articular cartilage and showed high intraobserver and interobserver reproducibility in detecting the presence or absence of a discernible banding pattern described as cartilage OCT form birefringence [5]. Bear et al. showed that the degree of OCT form birefringence correlated with polarized microscopy [24]. Furthermore, metabolic study of fresh human cartilage explants with and without OCT form birefringence suggest that loss of OCT form birefringence may be a marker for early cartilage degeneration. Articular cartilage explants were incubated in IGF-1, and proteoglycan synthesis was measured. Explants with no surface abnormality and a lack of form birefringence exhibited insensitivity to the anabolic effects of IGF-1 while those retaining form birefringence showed increased proteogy can synthesis in response to IGF-1 (*P* < .05). Both chondrocyte insensitivity to growth factors and microstructural loss of collagen organization are seen in the earliest stages of cartilage degeneration and therefore give support to OCT as a nondestructive imaging modality for early diagnosis of cartilage pathology [5, 7].

Han et al. sought to evaluate the utility and limitations of OCT by immediate, high-resolution microstructural analysis of articular repair tissue following allogeneic chondrocyte implantation without excising or sectioning the specimen in a mammalian animal model. Following chondral defect formation and chondrocyte implantation in the trochlear grove of a rabbit knee, the subsequent repair tissue was analyzed by arthroscopic surface imaging, OCT, and histology [25]. The authors found that OCT enabled the micro-structural evaluation of articular repair tissue and the detection of surface fibrillation, tissue hypertrophy, and cartilage integration similar to low power microscopy without damaging the repair. Most importantly, OCT was able to detect subsurface gaps between the repair tissue and native cartilage that were undetectable by arthroscopic assessment [25]. These results demonstrate that OCT is capable of providing an optical biopsy of articular repair cartilage without damaging the specimen, and suggest that, if incorporated into an arthroscope, it could potentially be used to evaluate articular cartilage repair *in vivo.*

**Figure 2:**
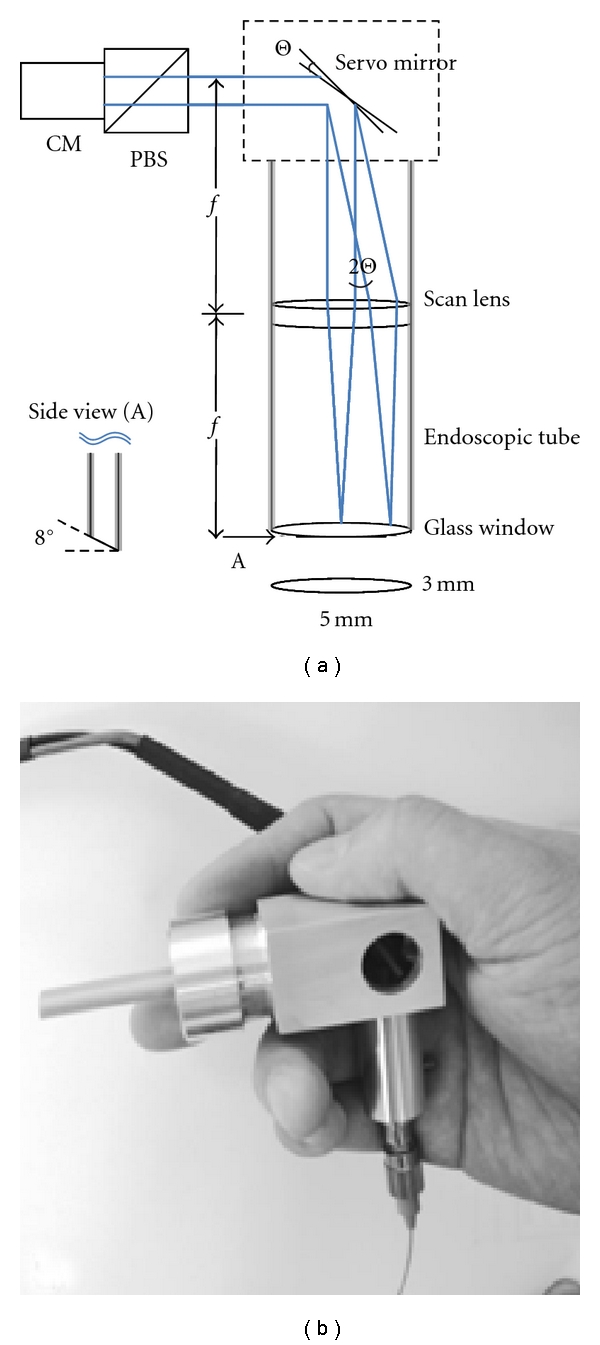
Arthroscopic OCT probe. (a) A schematic diagram of the OCT probe. (b) A photograph of the hand-held OCT arthroscope probe. PBS, polarization beam splitter; CM, fiber optic collimator.

Following these encouraging results, Pan et al. using fiber optic technology, described the use of a hand-held OCT probe capable of providing an optical biopsy of articular cartilage while fully immersed in saline during arthroscopy (Figure 2) [26]. The authors then evaluated the ability of the arthroscopic OCT probe to nondestructively detect microstructural cartilage changes as compared to histology in human cadaver knees [7]. The cartilage of human cadaver knees was graded both arthroscopically using OCT and then histologically using a Modified Mankin Structural Score following excisional biopsy. Using weighted Kappa statistics, the investigators found good agreement (*κ* = 0.80) between OCT and histology overall, but found substantial agreement (*κ* = 0.87) for specimens assigned a Modified Mankin score of 0–3 indicating improved diagnostic aptitude at the earlier stages of cartilage degeneration. 

**Figure 3:**
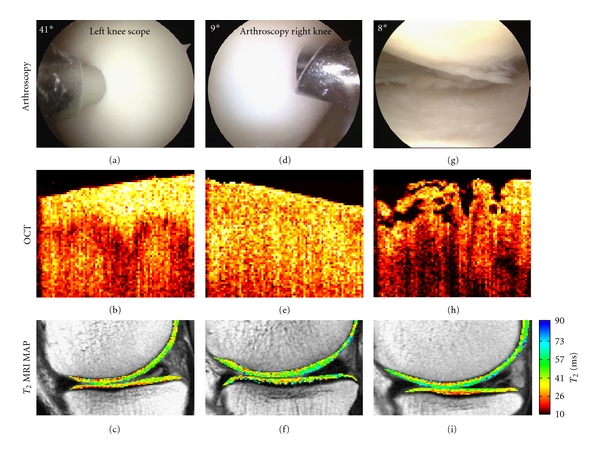
Representative images obtained during arthroscopy, optical coherence tomography (OCT), and magnetic resonance imaging (MRI) *T*_2_ mapping relaxation times. The higher the *T*_2_ relaxation time reflects a greater degree of hydration which may correlate with increased articular cartilage degeneration. ((a)–(c)) arthroscopically firm (a), OCT with birefringence (b), and MRI *T*_2_ mapping (c). ((d)–(f)), arthroscopically firm (d), OCT without birefringence (e), and MRI *T*_2_ map (f). ((g)–(i)), arthroscopic fissuring (g), OCT with surface fissuring and fibrillation (h), and MRI *T*_2_ map (i).

MRI is another nondestructive imaging modality with sequences such as *T*_2_ mapping that have been shown to be sensitive to collagen orientation and biomechanical integrity and is postulated to be dependent on collagen orientation and tissue hydration [27]. Experimentally, OCT was found to correlate with MRI *T*_2_ map and with progressive cartilage degeneration as determined by polarized microscopy [24]. In a Level 1 clinical diagnostic study, Chu et al. compared arthroscopic OCT, and high resolution 3 Tesla MRI *T*_2_ mapping against arthroscopy as the clinical standard in 30 human subjects undergoing arthroscopy for degenerative meniscus tears (Figure 3) [8]. When compared to arthroscopy, quantitative OCT was found to strongly correlate with arthroscopic grading (*R* = 0.85, *P* = .0002) while MRI *T*_2_ map did not. This correlation is important as OCT improves on conventional arthroscopy by high resolution imaging of subsurface as well as surface abnormalities and by contributing quantifiable data. MRI is a low resolution cross-sectional imaging modality and was unable to accurately diagnose subtle surface abnormalities found on arthroscopy. However, a correlation was found between superficial MRI *T*_2_ map and quantitative OCT likely because both metrics were based on cross sectional imaging data. This finding is important in supporting a diagnostic potential of MRI *T*_2_ map and other quantitative MRI technologies that are noninvasive and therefore can be more widely performed than arthroscopy or OCT. Currently, the clinical diagnostic potential of MRI *T*_2_ map and other MRI technologies for cartilage abnormalities is controversial in part due to the previously noted limitations of arthroscopy as a clinical standard. OCT provides quantifiable high-resolution cross-sectional data to improve on some of the shortcomings of conventional arthroscopy and was able to support the MRI *T*_2_ map findings. OCT is therefore shown to be an important translational clinical research tool, to assist in validating noninvasive but lower-resolution cross-sectional MRI technologies that may poorly correlate with conventional arthroscopy. 

Similar to arthroscopy, OCT provides diagnostic information in near real time. Acute articular cartilage injury following joint injuries such as anterior cruciate ligament tear and intra-articular fracture likely contribute to development of posttraumatic osteoarthritis. Often there are no recognizable surface abnormalities appreciated upon surgical intervention. The joint-injured patient represents a population at high risk for early disabling osteoarthritis who would benefit from diagnosis and treatment of cartilage injury and degeneration prior to the development of irreversible changes [3, 28]. We and others are currently studying potential chondroprotective agents that may improve cartilage survival after impact injury. In a study evaluating OCT and its ability to detect acute cartilage changes following impact injury, Bear et al. showed significant correlation between histology, chondrocyte death, and OCT grading following impact injury even at energies insufficient to fracture the articular surface (*R* ^2^ = 0.48, *P* < .001) [28]. In a pilot study of ACL-injured subjects, OCT was found to detect microscopic subsurface injuries that were predictive of MRI *T*_2_ map changes at 6 and 12 months following ACL reconstruction (unpublished data). These data further show a potential for OCT to nondestructively detect microstructural subsurface injuries to articular cartilage that were undetectable by conventional surface examination. 

## 5. Future Directions

The potential clinical implications of early diagnosis and staging of acute cartilage injury include supporting a clinical paradigm shift from viewing osteoarthritis as an untreatable degenerative condition to that of a potentially modifiable chronic disease process. Towards this end, laboratory studies show OCT can potentially provide microstructural information on cartilage health that can be used to improve the diagnosis and staging of early cartilage injury and degeneration [7, 24, 28]. Translational clinical studies support the use of OCT arthroscopically for these purposes [5, 8]. Recent technological developments include decreasing the size of the OCT probe to where it can be inserted through an 18 gauge needle, potentially making OCT evaluation of the cartilage an office procedure [29]. Recent studies additionally show OCT to be a powerful translational research tool in assessing the clinical utility of new MRI technologies for noninvasive early detection of cartilage injury and degeneration. Further study of OCT and related new technologies for assessment of articular cartilage will assist in the development of the clinical diagnostic power needed for implementation and evaluation of potential new treatment strategies to delay or prevent the onset of osteoarthritis.
